# The Effectiveness of Orofacial Myofunctional Therapy in Adults with Myogenous Temporomandibular Disorders: Insights from a Pilot Study

**DOI:** 10.3390/jcm14248718

**Published:** 2025-12-09

**Authors:** Paulina Czarnecka, Bartosz Bujan, Anna Maria Pekacka-Egli

**Affiliations:** 1 Institute of Linguistics, Faculty of Humanities, University of Silesia, ul. Uniwersytecka 4, 40-007 Katowice, Poland; 2 Neurorehabilitation, Klinik Lengg, 8008 Zurich, Switzerland; bartosz.bujan@kliniklengg.ch (B.B.); or annamaria.pekackaegli@fhnw.ch (A.M.P.-E.); 3 Department of Communicative Participation and Speech and Language Therapy, FHNW University of Applied Sciences and Arts, 4132 Muttenz, Switzerland

**Keywords:** orofacial myofunctional therapy, temporomandibular disorders, myofunctional disorders, orofacial dysfunction

## Abstract

**Background:** Temporomandibular disorders (TMDs) are increasingly understood within the biopsychosocial framework, which highlights the interplay of biological, psychological, and social factors in their onset and persistence. Within this context, orofacial myofunctional disorders (OMDs) represent a significant biological component, reflecting structural and functional disturbances of the orofacial system that may contribute to temporomandibular dysfunction. **Objectives:** This pilot study evaluated the effectiveness of orofacial myofunctional therapy (OMT) in improving functional parameters and reducing pain in adults with myogenous TMD accompanied by OMDs. **Methods:** In this prospective single-arm pilot study, twenty-five adults (aged 25–39 years) with myogenous TMD and coexisting OMDs, diagnosed according to DC/TMD criteria by a dentist trained in DC/TMD assessment and referred for the intervention, completed three biweekly OMT sessions. The therapy comprised myofascial release, oromotor exercises, functional retraining of breathing, chewing, and swallowing, as well as mandibular stabilization and dissociation exercises, complemented by home-based practice. Functional parameters—maximum mouth opening (MAX) and tongue mobility (TRMR-TIP, TRMR-LPS)—were measured before and after each session. Pain intensity (VAS) and quality of life (SF-36) were assessed at baseline and post-intervention. Data were analyzed using the Shapiro–Wilk test, paired t-test, and Wilcoxon signed-rank test. **Results**: Statistically significant improvements (*p* < 0.001) were observed across all evaluated parameters. Participants demonstrated increased maximum mouth opening and tongue mobility, along with decreased pain intensity and improved quality of life following the intervention. **Conclusions**: This pilot study provides preliminary evidence that short-term OMT can yield measurable functional improvements and pain reduction in adults with TMD and associated OMDs. These findings underscore the relevance of addressing orofacial myofunctional impairments as part of the biological dimension within the biopsychosocial model and support the integration of OMT into interdisciplinary TMD management.

## 1. Introduction

Temporomandibular joint disorders (TMDs) comprise a heterogenous group of conditions affecting the masticatory system, including the temporomandibular joints (TMJs), associated muscles, and the surrounding neural and connective structures [1,2]. Clinical manifestations typically include pain, joint sounds, abnormal jaw mobility and masticatory muscle tension [3,4]. According to the Diagnostic Criteria for Temporomandibular Disorders (DC/TMD), myogenous TMDs (M-TMDs) are classified by the pattern and distribution of pain elicited during examination, including myalgia, local myalgia, myofascial pain, and myofascial pain with referral [5,6]. Temporomandibular disorders (TMDs) are among the most prevalent musculoskeletal conditions, currently affecting approximately 34% of the population, with projections suggesting an increase to 44% by 2050 [7]. Their multifactorial etiology and increasing prevalence underline the clinical relevance of early, function-oriented management.

**Figure 1:**
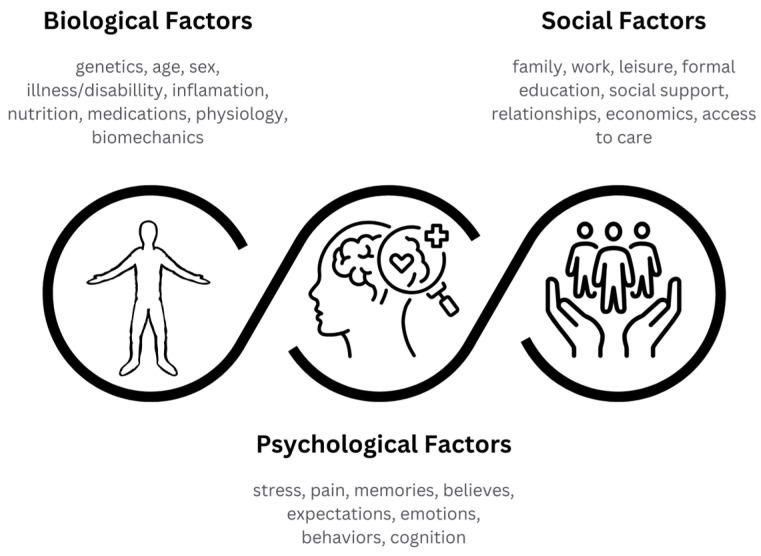
Biopsychosocial model of temporomandibular joint disorders (TMDs). The model illustrates the interaction of biological, psychological and social factors contributing to the onset and maintenance of temporomandibular dysfunction. Original figure by the authors, conceptually adapted from Engel’s biopsychosocial model [8].

The current understanding of TMDs is grounded in the biopsychosocial model, which integrates biological, psychological, and social dimensions of disease (Figure 1). Figure 1 illustrates how biological, psychological, and social factors interact in the onset and persistence of TMD. Psychological stress and parafunctional habits (e.g., clenching, bruxism) can increase muscular tension, while social and lifestyle components (e.g., occupational strain, dietary habits) influence compensatory oral behaviors.

Within this multidimensional framework, orofacial myofunctional disorders (OMDs) are recognized as a relevant biological component contributing to the onset and persistence of TMDs. OMDs involve structural and functional disturbances of the lips, jaw, tongue, and oropharynx that can influence or condition the normal development and function [9]. They manifest through dysfunctional respiration, atypical oral resting posture, impaired mastication and deglutition, and asymmetric muscular activity, all of which can disrupt the equilibrium of the stomatognathic system and contribute to musculoskeletal strain within the TMJ complex. In this study, OMDs were operationally defined based on dysfunctional orofacial resting posture, restricted tongue mobility (TRMR values below normative references), and impaired mastication or swallowing confirmed during structured clinical myofunctional assessment.

**Figure 2:**
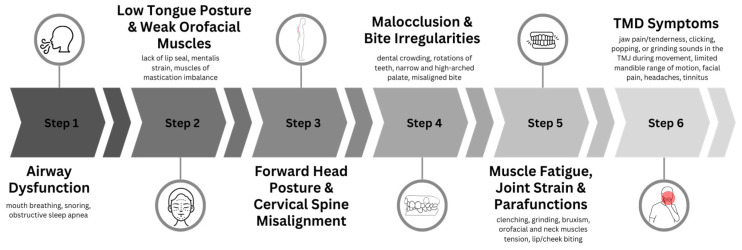
Hypothetical mechanism linking myofunctional disorders (OMDs) to temporomandibular disorders (TMDs).

OMDs often arise from maladaptive patterns such as chronic mouth breathing or insufficient nasal breathing, which can lead to altered craniofacial growth and malocclusion [9,10]. The relationship between OMDs and TMDs is illustrated in Figure 2. Physiologically, OMD and TMD are interrelated through compensatory muscle activity and altered proprioceptive feedback within the orofacial system. Dysfunctional tongue and jaw postures can increase tension in the elevator and suprahyoid muscles, modify mandibular kinematics and occlusal loading, and elevate temporomandibular joint stress. Persistent imbalance of orofacial muscle tone may disrupt the coupling among breathing, mastication, and swallowing, promoting myogenous pain and functional limitation.

Their multifactorial origin includes learned behaviors, anatomical deviations, genetic predispositions, and environmental factors [9,11,12]. OMDs have been associated with restricted tongue mobility (ankyloglossia), obstructive sleep apnea (OSA), bruxism, and postural alterations including forward head posture [13,14,15,16,17,18,19,20]. These dysfunctions necessitate targeted therapeutic interventions [20,21,22].

Orofacial myofunctional therapy (OMT) is a structured, exercise-based approach aimed at re-educating the neuromuscular patterns of the orofacial system to restore balanced muscle function and physiological resting posture. OMT may influence both peripheral and central neuromuscular processes, although causal mechanisms cannot be determined from this pilot study. Further neuroimaging research in this field is highly warranted.

**Figure 3:**
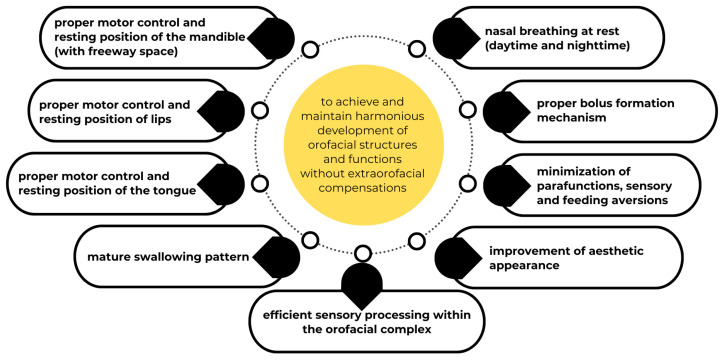
Goals of orofacial myofunctional therapy (OMT).

An overview of OMT objectives is presented in Figure 3.

Given the shared musculature and neural pathways involved in mastication, swallowing, and breathing, dysfunction in one component may influence the others. OMDs can therefore maintain or exacerbate TMD symptoms.

Previous studies have indicated that OMT may improve mandibular range of motion, tongue mobility, and pain perception, as well as alleviating otologic symptoms associated with TMD [23,24,25,26,27,28,29]. OMT thus represents a potential non-invasive, function-oriented approach that complements conventional dental, orthopedic, and physiotherapeutic interventions.

Despite growing clinical use of OMT, evidence supporting its effectiveness in adults with myogenous TMD remains limited. Most studies present substantial methodological variability and clear gaps that justify our pilot design. Existing OMT studies [23,24,26,27] include inconsistent protocols and typically omit components that are essential in myofunctional rehabilitation, such as orofacial awareness training, muscle–fascial relaxation work, and structured self-massage, all of which are integral to our protocol. These methodological gaps justify the need for exploratory pilot data to support future controlled research. Therefore, this pilot study sought to explore the potential impact of OMT on pain and function in adults with TMD. The present pilot study aimed to assess the effectiveness of structured OMT protocol in adults with myogenous TMD and coexisting OMDs. Specifically, it investigated changes in mandibular and tongue mobility, pain intensity and self-reported quality of life (QoL) following a short-term intervention. Based on this rationale, the null hypothesis was that there would be no significant pre- to post-intervention changes in mandibular mobility, tongue mobility, pain intensity or self-reported quality of life. The study further sought to provide preliminary evidence supporting the integration of OMT into interdisciplinary management of TMD within the biopsychosocial framework.

## 2. Materials and Methods

### 2.1. Study Design

This pilot study employed a prospective interventional design to evaluate the effects of structured orofacial myofunctional therapy (OMT) protocol on functional and subjective outcomes in adults with myogenous temporomandibular disorders (TMDs) and coexisting orofacial myofunctional disorders (OMDs). The study was conducted between July and October 2025 in private practice in Zabrze, Poland. Each participant completed three therapeutic sessions spaced two weeks apart. Measurements were obtained immediately before and after each session to capture both cumulative and session-specific effects.

### 2.2. Participants

Twenty-five adults (both men and women) aged 25–39 years were recruited. All participants had a confirmed diagnosis of myogenous TMD with a muscular pain component (ICD-9: 729.1-myalgia, localized myalgia, or myofascial pain) based on DC/TMD criteria, established by a dentist trained in DC/TMD assessment prior to referral, and presented with coexisting OMDs. The DC/TMD distinction between local myalgia, myofascial pain and myofascial pain with referral was applied during diagnostic classification. The presence of OMDs was verified using a structured clinical myofunctional assessment including evaluation of tongue mobility (TRMR), orofacial resting posture, mastication and swallowing, aligned with current international consensus recommendations. Exclusion criteria included active oral infections, recent maxillofacial surgery (within six months), initiation of orthodontic treatment during the study period, and systemic or neurological conditions that influence therapy outcomes. All participants provided written informed consent prior to study inclusion. The age range of 25–39 years was chosen to ensure relative homogeneity of musculoskeletal properties and to minimize confounding age-related differences in muscle tone and tissue elasticity.

### 2.3. Therapeutic Intervention

All participants received treatment according to an original OMT program developed by the authors to normalize muscle balance and functional coordination in the orofacial region. While the same structured therapeutic progression was applied to all participants, the intensity and complexity of exercises were individually adapted to neuromuscular capacity in accordance with established principles of myofunctional therapy. Each participant attended three therapy sessions over a four-week period (one session every two weeks). Each session lasted approximately 30 min and followed the same structured sequence. Exercise intensity and complexity were progressively adjusted according to individual performance. Home exercises were supported by written and video instructions, and compliance was verified through exercise logs reviewed at each session. The same licensed speech-language pathologist conducted all sessions to ensure methodological consistency. The intervention consisted of five integrated components:

1. Awareness and mindfulness training: Participants were educated on physiological versus dysfunctional orofacial patterns and introduced to breathing awareness and orofacial mindfulness, both during in-office sessions and at home.
2. Logopedic Myofascial Massage: Manual myofascial release techniques targeted soft-tissue restrictions in the shoulder girdle, neck, suprahyoid region, and masticatory and facial muscles. The logopedic myofascial massage used in this study represents an adaptation of classical myofascial release techniques for the orofacial region, designed and applied by a speech-language pathologist trained in manual therapy.
3. Oromotor training: passive, active, and resistance exercises were applied to enhance range, precision, and strength of movements of the lips, tongue, and mandible. Exercises were adapted to individual functional capacities.
4. Functional Re-education: Training focused on restoring physiological orofacial functions, including nasal–diaphragmatic breathing (Buteyko method), mandibular stabilization and dissociation, bilateral mastication, physiological swallowing and physiological resting posture of the tongue, lips, and mandible with freeway space [30,31]. These components were introduced in a progressive sequence, prioritizing breathing normalization before masticatory and swallowing re-education to support coherent neuromuscular integration.
5. Habituation and self-therapy: Participants received an individualized home program to reinforce newly acquired motor patterns through daily self-massage and consolidation exercises. The habituation phase lasted approximately two weeks after completion of the in-office sessions. Participants were contacted once by phone to verify stability and continued adherence. Adherence was monitored through daily exercise logs and self-reports collected at each session. Participants were instructed to record frequency and duration of home practice to ensure consistency in exercise performance. The applied OMT protocol extended the established approach proposed by de Felício et al. which provide structured frameworks for orofacial myofunctional retraining [23].

### 2.4. Measurement Protocol

**Figure 4:**
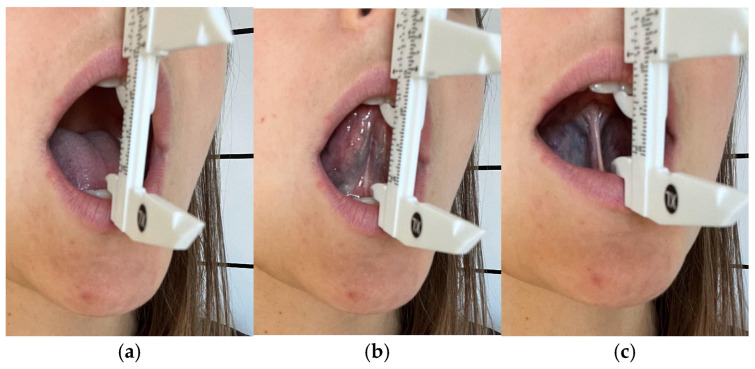
(**a**) Measurement of MAX; (**b**) Measurement of tongue tip to the incisive papilla—TIP; (**c**) Measurement of tongue in lingual-palatal suction—LPS.

Outcome measures included tongue and mandibular mobility, pain intensity, and self-reported quality of life. All assessments were conducted by the same examiner under standardized conditions:

- Maximum mouth opening (MAX): The interincisal distance (mm) was measured with a caliper before and after each therapy session. (Figure 4a). Maximum mouth opening was measured actively. Participants were instructed to stop before pain occurred to ensure measurement safety.
- Tongue Range of Motion Ratio (TRMR): Two indices were recorded—TRMR-TIP (tongue tip to incisive papilla: Figure 4b) and TRMR-LPS (lingual-palatal suction: Figure 4c)—following the methods of Zaghi et al. [32]. Values were expressed in millimeters and represented the ratio between maximum mouth opening and tongue elevation tasks. Each measurement was repeated three times with participants seated upright; the mean value was used for analysis. Intraclass correlation coefficients (ICC) were calculated, indicating excellent reliability (ICC > 0.90).
- Visual Analogue Scale (VAS): Participants rated intensity on a 10-point scale at base and after the third session.
- SF-36 Questionnaire [33]: Self-reported quality of life was evaluated before the first and after the third therapy session. Lower scores indicated a better perceived quality of life. In this pilot context, the global SF-36 score was used to provide an overall proxy of health-related quality of life while minimizing respondent burden and enabling preliminary feasibility assessment.

All measurements were performed twice and averaged. Intra-rater reliability, assessed by repeated measurements in five randomly selected participants, exceeded 95%.

### 2.5. Statistical Analysis

All analyses were performed using Statistica 13.3 (StatSoft Inc., Tulsa, OK, USA). Normality was verified with the Shapiro–Wilk test. For normally distributed data, paired *t*-tests were applied; for non-normal data, Wilcoxon signed-rank tests were used. Effect sizes (Cohen’s d) and 95% confidence intervals (CI) were calculated and interpreted using thresholds for TMD research (small = 0.10, medium = 0.30, large = 0.70). CI interpretation followed established recommendations [34]. Significance was set at *p* < 0.05.

## 3. Results

All 25 participants completed the three sessions and associated assessments. Statistical analyses were associated with improvements across all measured parameters.

### 3.1. Changes in Mandibular Mobility

**Figure 5:**
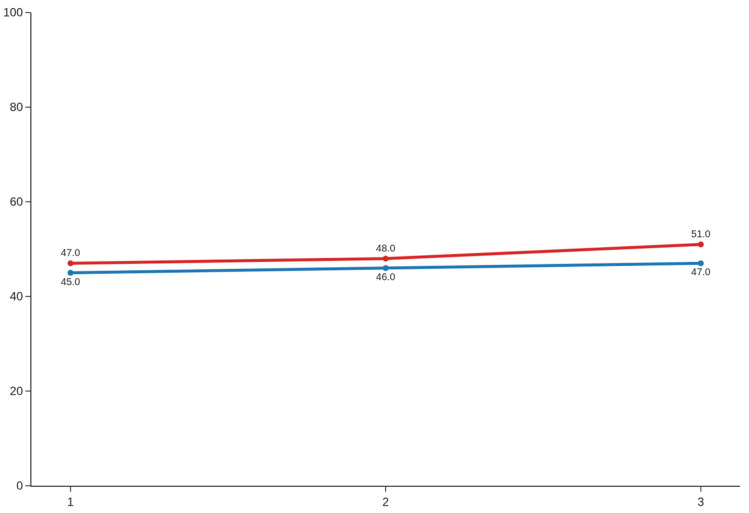
MAX [mm] along 3 sessions; blue line—before, red line—after.

A progressive increase in maximum mouth opening (MAX) was observed over the course of therapy. Median values rose from 45 mm before the first session to 47 mm after session one, with continued improvement across subsequent sessions (Figure 5). Analyses of maximum jaw opening across the three therapy sessions revealed consistently large and statistically significant effects (*p* < 0.0001). Because the data did not meet normality assumptions for any of the sessions, all comparisons were conducted using the Wilcoxon signed-rank test. The first therapy session showed a substantial increase in maximum jaw opening, with an effect size of d = 1.20 (95% CI: 0.79–1.62). An even larger effect was observed following the second therapy session (d = 1.39; 95% CI: 0.98–1.80), representing the most pronounced improvement in the series. The third session also yielded a large effect, with d = 1.16 (95% CI: 0.75–1.58). Collectively, these findings indicate robust and clinically meaningful gains in maximum jaw opening across all stages of therapy.

As objective myofunctional biomarkers remain limited, quantifiable measures such as mandibular range of motion continue to serve as useful indirect indicators of short-term neuromuscular responsiveness. Even minimal changes may reflect early adaptive processes within the masticatory muscles, especially when assessed systematically across multiple sessions. This pattern suggests potential functional enhancement in mandibular mobility. In individuals with myogenous TMD, even modest increases in maximum mouth opening are considered clinically meaningful because improvements typically reflect reduced pain-related guarding and enhanced functional mandibular mobility.

### 3.2. Changes in Tongue Mobility

**Figure 6:**
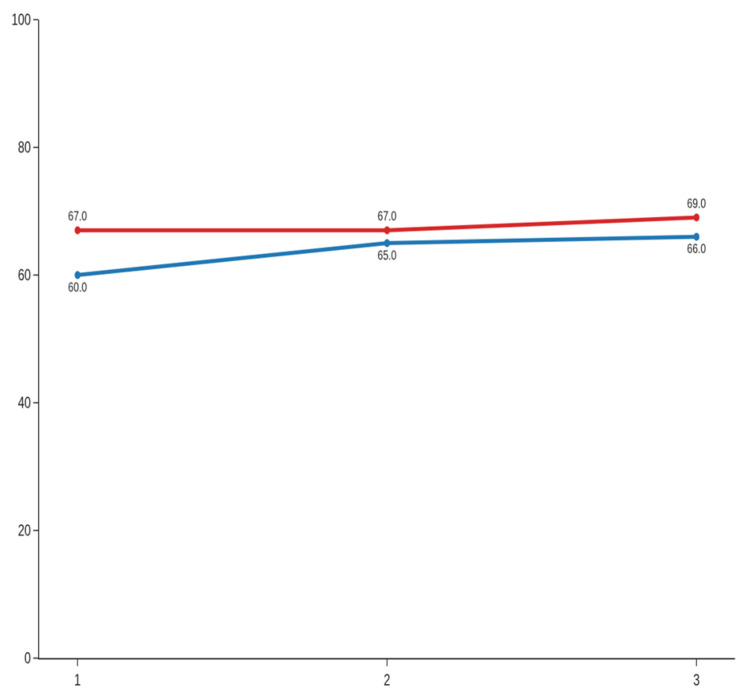
TRMR-TIP [%] along 3 sessions; blue line—before, red line—after.

**Figure 7:**
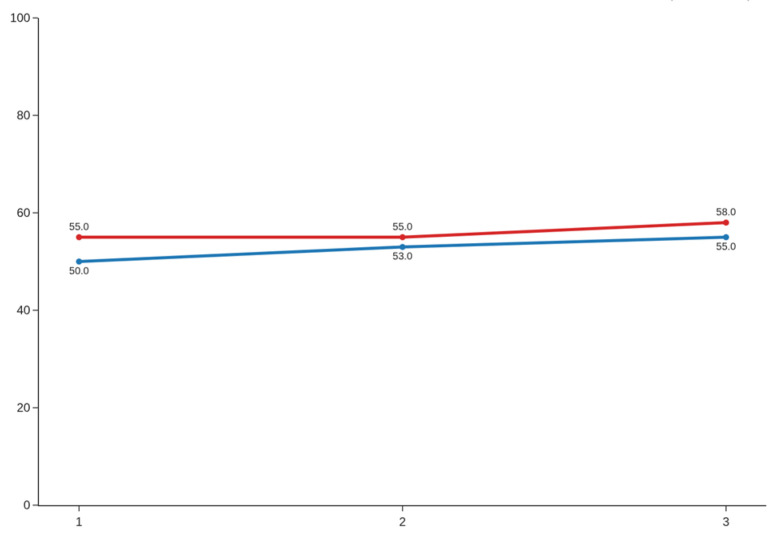
TRMR-LPS [%] along 3 sessions; blue line—before, red line—after.

Significant improvements were also noted in tongue range of motion. For TRMR-TIP, paired t-tests indicated statistically significant changes at all time points, with medium effect sizes: d = 0.76 (95% CI: 0.35–1.18) for session 1, d = 0.57 (95% CI: 0.16–0.98) for session 2, and d = 0.53 (95% CI: 0.12–0.95) for session 3 (Figure 6). Even larger effects were observed for TRMR-LPS. Although the first comparison required a Wilcoxon signed-rank test due to non-normality, the resulting effect size remained substantial (d = 1.14; 95% CI: 0.73–1.55). Significant improvements were also noted for session 2 and 3, with paired *t*-tests yielding large effect sizes of d = 0.96 (95% CI: 0.55–1.38) and d = 1.16 (95% CI: 0.75–1.57), respectively (Figure 7). Overall, the data indicate robust and consistent enhancement in tongue mobility across sessions, particularly for the lingual–palatal suction measure.

These results demonstrate a consistent session-by-session improvement in lingual mobility and functional control, without evidence of regression between sessions. These mobility improvements may reflect reduced pain-related guarding and enhanced motor control, even if patients do not yet perceive changes in maximum opening as functional improvements.

### 3.3. Pain Intensity and Quality of Life

Pain intensity, measured by the Visual Analogue Scale (VAS), decreased systematically throughout the study period, while SF-36 scores reflected improved self-reported quality of life. For all comparisons, *p* < 0.001. Table 1 and Table 2 summarize the findings. The data has been updated and completed as requested. The reduction in VAS scores following OMT was not only statistically significant but also clinically substantial. The paired-samples effect size was exceptionally large (Cohen’s d = −2.62, 95% CI [−3.77, −2.07]), indicating a robust and consistent shift toward lower pain intensity across participants. The corresponding effect size expressed as r (0.94) likewise reflects a very strong association between the intervention and the observed change. Taking together with the descriptive statistics shown in Table 1, these results suggest that the analgesic response to OMT was both pronounced and broadly shared within the cohort. A similarly strong pattern emerged for the SF-36 scores, where improvements in self-reported quality of life were marked by an even larger effect size. The magnitude of change (Cohen’s d = −3.71, 95% CI [−5.23, −2.93]) underscores an intervention effect of exceptional strength, with the effect size r reaching 0.97. This indicates that the post-treatment shift was not only statistically reliable but also pervasive across individuals. In line with the descriptive trends presented in Table 2, the data point to a meaningful enhancement in perceived functional well-being following OMT, consistent with a substantial therapeutic impact.

The results demonstrate statistically significant gains in mandibular opening and tongue mobility following OMT accompanied by marked reductions in pain and perceived well-being. The magnitude and consistency of these changes support the clinical relevance of the intervention and reduce the likelihood that the outcomes resulted from random variation.

## 4. Discussion

This pilot study suggests that a short-term, structured orofacial myofunctional therapy (OMT) program may be associated with functional and symptomatic improvements in adults with myogenous temporomandibular disorders (TMD) and coexisting orofacial myofunctional disorders (OMD). OMT appears to exert a multidimensional influence through neuromuscular rebalancing and improved integration of stomatognathic subsystems. Medium and large effect sizes were primarily observed for tongue mobility and pain reduction, suggesting that these domains may be most sensitive to short-term OMT. The consistent upward trends in mandibular and lingual mobility after each session may indicate neuromuscular adaptations, although causal inferences cannot be drawn in the absence of a control group. These results are comparable in magnitude to improvements reported in physiotherapy-based interventions for TMD, but OMT differs by focusing on neuromuscular re-education rather than joint mobilization or passive tissue manipulation. This distinction underscores the functional nature of OMT and its potential to complement manual therapies within multimodal programs. The observed effects should be interpreted with caution due to the limited sample size and absence of randomization.

The findings align with previous reports by de Felicio et al. [23,24] and Melis et al. [25], who documented similar gains in mandibular function and pain reduction following structured orofacial exercises. Improvements in tongue mobility are particularly noteworthy, given that limited lingual range of motion and atypical tongue posture are frequently implicated in maintaining TMD-related dysfunction. The results reinforce the hypothesis that OMDs not only coexist with TMDs but may act as perpetuating factors within the pathophysiological mechanism. Integrating consistent cognitive-behavioral and functional-behavioral strategies may further support sustainable neuromuscular re-education as long-term adherence within interdisciplinary care models.

From the clinical standpoint, the integration of OMT into interdisciplinary care appears justified. Addressing muscular imbalance, atypical postures, and dysfunctional breathing or swallowing patterns provides a biological foundation for sustainable recovery. Combining OMT with dental, orthopedic, and physiotherapeutic approaches may enhance functional restoration and prevent relapses by targeting both structural and neuromuscular dimensions. This integrative perspective corresponds with the biopsychosocial model, emphasizing interaction among physiological, behavioral, and environmental components. In practical terms, OMT can be integrated into interdisciplinary orofacial pain clinics, dental rehabilitation programs, or speech therapy settings. Effective implementation requires professional training in orofacial myofunctional assessment, coordinated referral pathways, and structured collaboration between speech-language pathologists, dentists, and physiotherapists. The functional gains observed may reflect sensorimotor recalibration involving both peripheral and central mechanisms. Neuroimaging studies have demonstrated cortical plasticity in orofacial motor and somatosensory cortices following targeted oral exercises, supporting the hypothesis of central adaptation through repeated neuromuscular activation [35].

Several limitations must be acknowledged. The absence of a control group prevents exclusion of placebo or other non-specific treatment effects, and the same clinician conducted both therapy and assessments, which may introduce measurement bias. No blinding procedures were implemented, and a Hawthorne effect related to participant motivation cannot be excluded. The short observation period does not allow conclusions regarding long-term stability of the outcomes, and the homogeneous, relatively young sample limits generalizability. Applying DC/TDM classification, including Axis I and Axis II, is essential since psychosocial factors may influence therapeutic outcomes [5]. Furthermore, the single-arm design prevents differentiation of specific contributions of the myofascial massage component versus the functional exercise component. Future research should therefore adopt randomized controlled designs with larger and more diverse samples, follow-up measurements, and objective measures such as surface electromyography or motion-tracking analysis to better elucidate underlying neuromuscular mechanisms. Standardized, evidence-based OMT protocols will also be essential to enable replication and strengthen evidence synthesis across studies.

## 5. Conclusions

This pilot study demonstrated that a brief course of structured orofacial myofunctional therapy (OMT) can yield clinically and statistically significant improvements in tongue and mandibular mobility, accompanied by reduced pain intensity and enhanced quality of life in adults with temporomandibular disorders (TMDs) and coexisting orofacial myofunctional disorders (OMDs). These findings highlight the relevance of neuromuscular re-education within the orofacial system as part of a comprehensive, interdisciplinary treatment approach. Orofacial myofunctional disturbances should be regarded as meaningful biological contributors within the biopsychosocial framework of TMD. Their systematic identification and targeted management can complement conventional dental, physiotherapeutic, and behavioral strategies, potentially enhancing functional recovery and patient well-being.

Further controlled and longitudinal studies are required to confirm the durability of these outcomes, define standardized therapeutic protocols, and explore the physiological mechanisms underlying the efficacy of OMT in temporomandibular rehabilitation. Establishing evidence-based clinical guidelines will be essential for integrating OMT into mainstream interdisciplinary care for TMD. The present results emphasize that restoration of neuromuscular function, rather than symptomatic relief alone, should be regarded as a primary therapeutic goal in TMD rehabilitation.

## Figures and Tables

**Table 1:** VAS scores before and after OMT.

Measurement	Median	Minimum	Maximum	SD
VAS before OMT	7	5	9	1.15
VAS after OMT	4	1	7	1.38

**Table 2:** SF-36 scores before and after OMT.

Measurement	Median	Minimum	Maximum	SD
SF-36 before OMT	28	21	47	6.83
SF-36 after OMT	19	13	38	6.83

## Data Availability

Relevant data underlying the findings of this study can be obtained from the corresponding author, Paulina Czarnecka, upon reasonable request.

## Abbreviations

The following abbreviations are used in this manuscript:

TMD	Temporomandibular Disorders
OMD	Orofacial Myofunctional Disorders
OMT	Orofacial Myofunctional Therapy
TRMR	Tongue Range of Motion Ratio
TIP	Tongue to Incisive Papilla
LPS	Lingual Palatal Suction
MAX	Maximum mouth opening
VAS	Visual Analogue Scale
